# Kaempferol Identified by Zebrafish Assay and Fine Fractionations Strategy from *Dysosma versipellis* Inhibits Angiogenesis through VEGF and FGF Pathways

**DOI:** 10.1038/srep14468

**Published:** 2015-10-08

**Authors:** Fang Liang, Yuxiang Han, Hao Gao, Shengchang Xin, Shaodan Chen, Nan Wang, Wei Qin, Hanbing Zhong, Shuo Lin, Xinsheng Yao, Song Li

**Affiliations:** 1 Key Laboratory of Chemical Genomics, School of Chemical Biology and Biotechnology, Peking University Shenzhen Graduate School, Shenzhen, Guangdong, 518055, China; 2 Institute of Traditional Chinese Medicine & Natural Products, College of Pharmacy, Jinan University, Guangzhou, Guangdong, 510632, China; 3 Department of Biology, South University of Science and Technology of China, Shenzhen, 518055, China.; 4 Department of Molecular, Cell, and Developmental Biology, University of California, Los Angeles, Los Angeles, CA 90095, USA

## Abstract

Natural products are a rich resource for the discovery of therapeutic substances. By directly using 504 fine fractions from isolated traditional Chinese medicine plants, we performed a transgenic zebrafish based screen for anti-angiogenesis substances. One fraction, DYVE-D3, was found to inhibit the growth of intersegmental vessels in the zebrafish vasculature. Bioassay-guided isolation of DYVE-D3 indicates that the flavonoid kaempferol was the active substance. Kaempferol also inhibited the proliferation and migration of HUVECs *in vitro*. Furthermore, we found that kaempferol suppressed angiogenesis through inhibiting VEGFR2 expression, which can be enhanced by FGF inhibition. In summary, this study shows that the construction of fine fraction libraries allows efficient identification of active substances from natural products.

Natural products have been recognized as an invaluable resource for the isolation of therapeutic substances towards applications in human diseases. It contains vast and heterogeneous components that are regarded as better candidates to interact with biological macromolecules (usually proteins) than synthetic compounds due to evolutionary pressures^1^. Studies of natural products have led to the discovery of numerous drugs. For example, from January 1981 to December 2010, about 50% of small-molecule drugs were classified as naturally derived or inspired^2^,^3^. Typically, the strategy to isolate active natural products begins with an assay on crude extracts followed by bioassay-guided isolation. This strategy, however, tends to neglect rare components that are biologically active due to reciprocal interferences among many chemicals in the crude extracts.

To overcome the above disadvantages, we developed an alternative strategy beginning with the fine fractionation of the natural products. In this strategy, the crude extract is separated into a series of fine fractions through several established chromatographic methods. The number of fine fractions from a natural product varies, depending on the throughput of the adopted isolation method, the demand for the scale of the assay, and the amount of starting crude extract.

We chose zebrafish model to screen a library of fine fractions that were isolated from traditional Chinese herbal medicines. The rapid and easy to visualize development of the zebrafish embryo provides detailed morphological information on multiple traits (body axis, brain, eye, ear, heart, etc.). By using tissue–specific fluorescently labelled transgenic lines, many internal organs (liver, pancreas, intestine, pituitary, blood vessel,*etc*.) can also be examined. Furthermore, zebrafish offers the advantage of studying a system in an *in vivo* context, thereby allowing the elimination of substances with undesirable toxicity^4^. As a whole–organism screening system, zebrafish has made notable contributions in the identification of biologically active substances from both natural product and synthetic libraries^5^,^6^,^7^. Vascular development in zebrafish embryos has been well characterized^8^,^9^. In the trunk region, the vascular intersegmental vessels (ISVs) have a stereotyped pattern and are therefore well suited for angiogenic assays. This pattern can be directly visualized using the endothelial cell specific transgenic zebrafish line *Tg(kdrl:GRCFP)*^*zn1*^ in which GFP is under the control of vascular endothelial growth factor receptor 2 (VEGFR2, also known as *kdrl* or *flk*, Entrez Gene ID: 796537) promoter^10^. Utilizing this line allows for a quick, easy, and continuous observation of the vasculature in live embryos under a fluorescence microscope.

Angiogenesis is a necessary process for tumour growth, invasion and metastasis. Hence, approaches targeting angiogenesis have been actively pursued in cancer therapy options^11^,^12^,^13^. Tumour cells influence angiogenesis by releasing proangiogenic molecules such as vascular endothelial growth factor (VEGF) and basic fibroblast growth factor (bFGF)^14^,^15^. VEGF and FGF signalling are two important factors involved in tumour angiogenesis through downstream activation of the PI3K-Akt and Mek-Mapk-Erk pathways. These factors are also important in regulating proliferation, differentiation and survival of tumour cells^16^,^17^.

In this report, we describe a pilot screen of 504 fractions from nine traditional Chinese medicine (TCM) plants on *Tg(kdrl:GRCFP)*^*zn1*^ transgenic zebrafish for anti–angiogenesis substances. We identified kaempferol as a functional angiogenesis inhibitor from *Dysosma versipellis* (*D. versipellis*), a perennial herb belonging to the family *Berberidaceae*. The rhizome and radix of *D. versipellis* have been used as TCM to reduce fever, detoxify the body, reduce pain as well as treat viral infections and solid tumours. *D. versipellis* along with another herb *D. pleiantha* were first recorded in *Shennong’s Classic of Materia Medica* as “Gui Jiu” and have been used as a medicine in China over thousands of years^18^. We show that kaempferol inhibits the proliferation and migration of vascular endothelial cells through the suppression of VEGF and FGF signalling pathways.

## Results

### Preparation of fine fraction library and screening based on zebrafish model

**Figure 1:**
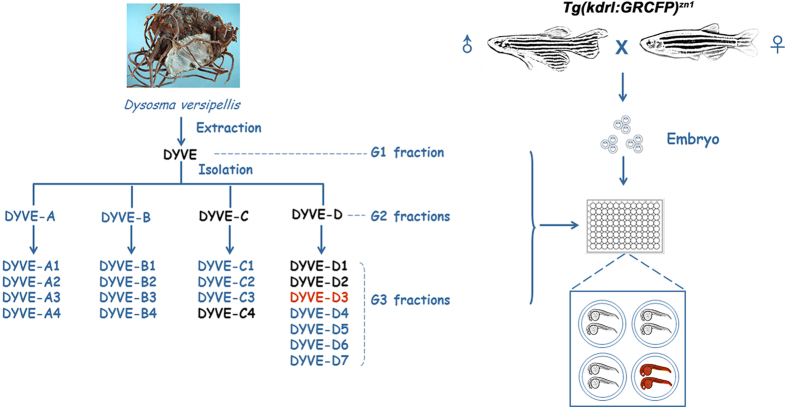
Schematic chart showing preparation of fine fractions from *D. versipellis* and zebrafish screening. The fractions marked with black caused severe embryonic defect but did not inhibit the outgrowth of ISVs. The fractions marked with blue did not cause a defective phenotype and resulted in normal embryos. The fraction DYVE-D3 marked with red was a positive hit, which inhibited the outgrowth of ISVs specifically. The zebrafish marked with red were screened out for inhibition of ISVs.

TCM is a rich natural resource for drug discovery^19^,^20^. We generated a library consisting of 504 fine fractions extracts from nine different TCM plants (Supplementary Table S1), following a three- or four-step procedure. For *D. versipellis,* three steps were followed to prepare G1–crude extract, G2–column chromatography fractions from G1, and G3–further separated fractions by column chromatography from G2 (Fig. 1).

Following fractionation, the library was screened using the transgenic zebrafish line *Tg(kdrl:GRCFP)*^*zn1*^, in which endothelial cells were specifically labelled by GFP^10^, to observe anti–angiogenic effects. Embryos were loaded into 96-well plates and fine isolated fractions were added to the embryos at about 6 hours post fertilization (hpf) at a final concentration according to Supplementary Table S1. At 30 hpf and 48 hpf, treated embryos were examined under a fluorescence microscope. The length of the ISVs was used as a measurement to evaluate angiogenesis, while other traits, such as body axis, brain morphology, and heart rate, were also examined.

**Figure 2:**
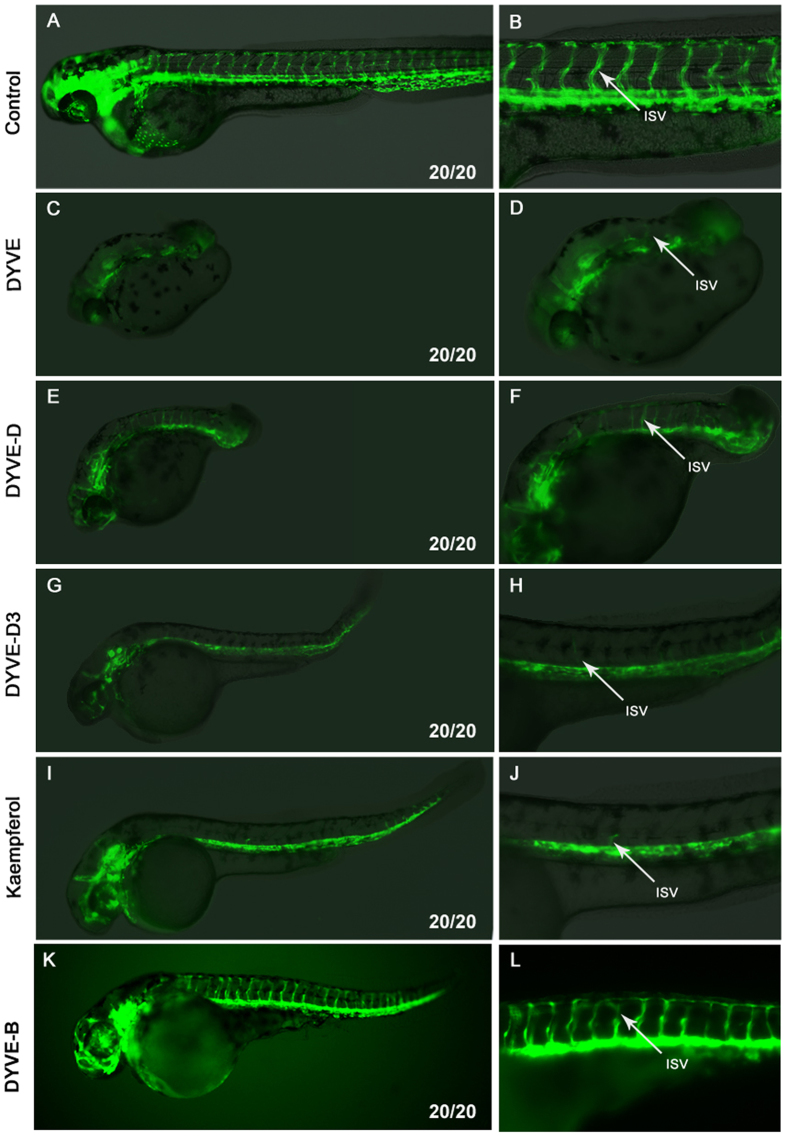
Fraction DYVE-D3 and kaempferol inhibited the outgrowth of ISVs. Embryos at 48 hpf anterior is to left and dorsal is to top. Right column is enlargement of left column. (**A,B**), control group, ISV fully developed. (**C,D**) DYVE group, embryonic trunk formation was impaired and the head was small, while the ISVs were still present. (**E,F**) DYVE-D group, the embryonic trunk was very short, and ISVs developed. (**G,H**) DYVE-D3 group, the embryo developed slower than the control, but did not have strong defects. Outgrowth of ISV was inhibited. (**I,J**) kaempferol group, embryos developed slower than control, but were mostly normal. Outgrowth of ISVs was inhibited. (**K,L)** DYVE-B group, the embryos were slightly abnormal on the morphology, but ISVs appeared normal. White arrows point to ISV. Phenotype ratio is showed in the lower right corner. The working concentration of DYVE, DYVE-D, DYVE-D3 and DYVE-B was based on Supplementary Table S1, and kaempferol group was 40 μM.

From the 504 fractions, DYVE-D3, a G3 fraction isolated from *D. versipellis*, was found to specifically inhibit the growth of ISVs with minimal toxicity (Fig. 2G,H). Careful analysis of the screen revealed that none of the G1 and G2 fractions of *D. versipellis* inhibited the growth of ISV (Fig. 1). The G1 fraction of DYVE caused severe embryonic defects. In addition to defect in ISVs, posterior trunk formation was also impaired (Fig. 2C,D). Among the four G2 fractions of *D. versipellis*, two fractions (DYVE-A and DYVE-B) had no effects (Fig. 2K,L). The other two fractions (DYVE-C and DYVE-D) caused embryonic defects and a high fatality rate. The embryos that survived had a short posterior trunk, with ISVs still present but not intact (Fig. 2E,F). These data indicate that these two fractions were less toxic than the G1 fraction. Among the nineteen G3 fractions of *D. versipellis*, only DYVE-D3 inhibited the growth of ISVs (Fig. 2G,H). This result indicates that our strategy is effective in both the elimination of toxic interferences co-existing in the crude extract and the detection of the less abundant bioactive substances that could have been missed in a classical screening strategy using crude extracts.

### Bioassay-guided isolation of kaempferol as the active compound in fraction DYVE-D3

**Figure 3:**
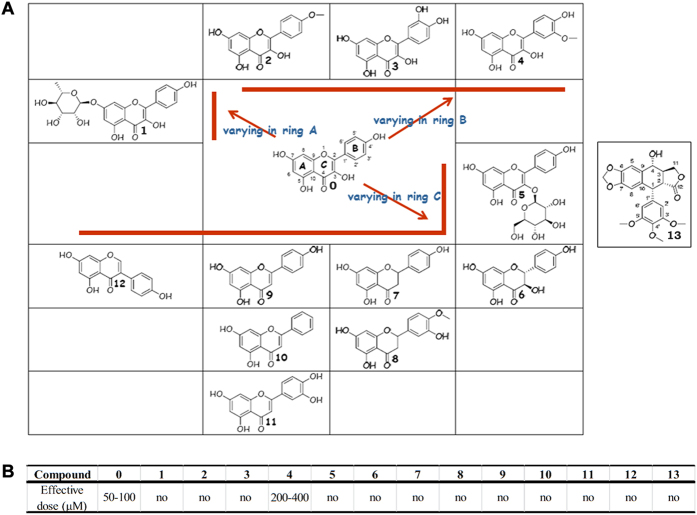
Antiangiogenic activity and structures of compounds 0 to 13. (**A**) compound **0** is kaempferol, which is the antiangiogenic substance in the fraction DYVE-D3. Compounds **1–12** are kaempferol analogues (ordered by structural variation of ring (**A**–**C**)) taken from the natural products library that was established by us. Compound **13** is podophyllotoxin, which is abundant in *D. versipellis* and has strong embryonic toxicity. (**B**) the effective dose in zebrafish compounds **0–13**.

The fraction DYVE–D3 was shown to contain only one bioactive compound, kaempferol, which is a common flavonoid present in a number of daily dietary plants, including tea leaves and strawberries^21^,^22^. Using pure kaempferol (≥97%) at 40 μM showed that it had the same anti-angiogenic activity on zebrafish embryos as the DYVE-D3 fraction (Fig. 2I,J). There are many kaempferol analogues in nature and we tested several of them (Compounds **1–12**, Fig. 3A) for their anti-angiogenic activity on the transgenic zebrafish embryos (Fig. 3B). Interestingly, only one analogue, compound **4**, inhibited angiogenesis at a higher dose. This finding demonstrates that the anti-angiogenic activity of kaempferol is of high specificity; therefore, subtle changes in structure may lead to a decrease or loss of activity.

### Anti-angiogenic activity of kaempferol on human umbilical vein endothelial cells (HUVECs)

**Figure 4:**
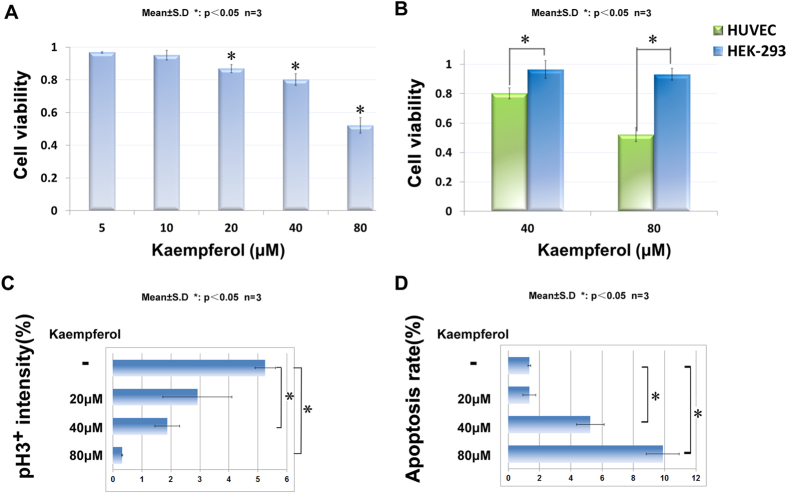
Kaempferol selectively inhibited mammalian endothelial cells and affected cell proliferation and apoptosis process. (**A**) inhibition of HUVECs viability by kaempferol in a dose-dependent manner. (**B**) kaempferol inhibited HUVECs more effectively than HEK-293 cells. HUVECs and HEK-293 cells were treated with different concentrations of kaempferol for 24 hours. Cell viability was quantified by MTT assay. (**C,D**) statistics of pH3/DAPI and TUNEL/DAPI double staining in HUVECs after 48 hours treatment with different concentration of kaempferol. The pattern is representative of three similar results. *P < 0.05 versus control group. Scale bar, 100 μm.

The process of angiogenesis consisting of cell proliferation, migration, and alignment to form tubular structures is conserved from mammals to lower vertebrates. To confirm the inhibitory effects of kaempferol in a mammalian system, we treated HUVECs with kaempferol for 24 hours, followed by a viability test using the MTT cell proliferation assay. As shown in Fig. 4A, the HUVECs cultured with kaempferol exhibited a viability reduction in a dose-dependent manner: 87% cell viability at 20 μM and 52% cell viability at 80 μM kaempferol. To investigate whether kaempferol is more specific to inhibit endothelial cells, we compared the sensitivity of HUVECs and HEK-293 (kidney cells) using the cell viability assay. As shown in Fig. 4B, HEK-293 cells were relatively insensitive to kaempferol. At 80 μM, the viability of HEK-293 cells was 93% while HUVECs displayed a viability of 52%, suggesting that kaempferol is more selective in inhibiting endothelial cells. Further study showed that the rate of cell proliferation was reduced significantly at 40 μM and 80 μM as revealed by immunostaining for phospho-histone H3 (Fig. 4C). Meanwhile, Terminal-deoxynucleotidyl transferase mediated nick end labelling (TUNEL) assay revealed that apoptosis was increased under the same condition (Fig. 4D). These findings demonstrate that kaempferol selectively inhibits endothelial cell viability through suppressing mitosis and promoting apoptosis.

**Figure 5:**
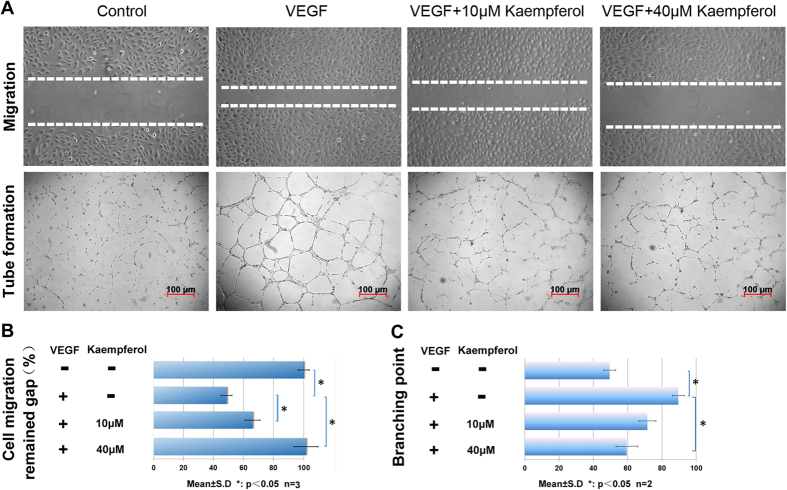
Kaempferol inhibits VEGF-induced migration and tube formation of HUVECs. (**A**) upper row, kaempferol inhibited VEGF-induced HUVECs migration in wound healing assay. HUVECs were allowed to grow into full confluence in 24–well plates. Cells were wounded with a pipette and treated with or without 40 ng/mL VEGF and different concentrations of kaempferol in ECM supplemented with 0.5% (v/v) FBS. Compared to control group, 40 μM kaempferol blocked induction by VEGF. Bottom row, kaempferol inhibited VEGF-induced HUVEC tube formation. (**B,C**) quantification of the results in (**A**). Image J was applied for statistical branching point numbers and the distance of remained gap. Scale bar, 100 μm. *P < 0.05 versus control group.

Next, we inspected the effect of kaempferol on migration of HUVECs using a scratch-wound assay. As shown in Fig. 5A (upper row) and 5B, kaempferol significantly inhibited VEGF-induced migration at 10 μM and 40 μM. Next, we used a Matrigel assay to test the effects of kaempferol on the tubular structure formation of HUVECs. In this system, when HUVECs were placed on the growth factor-reduced Matrigel in low-serum media, there was little tube formation. However, HUVECs tended to align and form tube-like structures after VEGF induction (Fig. 5A, bottom row). The ability of HUVECs to form tubular structures was assessed in the presence or absence of different concentrations of kaempferol. As shown in Fig. 5C, the tube branching point of kaempferol and VEGF co-treatment groups was less compared to that of the VEGF only treated group. Overall, these results show that the antiangiogenic activity of kaempferol is conserved in the mammalian system.

### Kaempferol suppressed angiogenesis by inhibiting VEGF and FGF pathways

Luo *et al.* investigated the antiangiogenic properties of kaempferol in human ovarian cancer cells and suggested that kaempferol inhibits angiogenesis by down-regulating levels of VEGF and HIF-1α^23^. Several studies have shown that both FGF and VEGF stimulate endothelial cell proliferation and differentiation through activation of the Erk1/2, Akt, and p38 mitogen-activated protein kinases (MAPK)^24^,^25^. *In vivo*, the growth factors VEGF and FGF exert a combined effect on both angiogenesis and the maturation of blood vessels^26^. To further understand the mechanism of kaempferol on angiogenesis, we analysed its function on VEGF and FGF signalling pathways.

**Figure 6:**
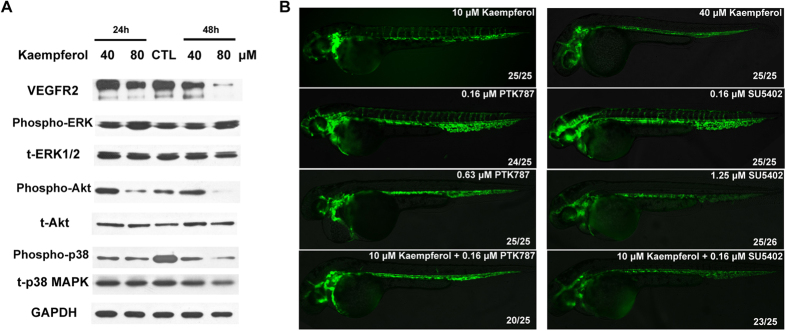
Kaempferol blocked PI3K/Akt and p38 MAPK *in vitro* and had a synergistic effect with PTK787/SU5402 on VEGF and FGF pathways *in vivo*. (**A**) different concentrations of kaempferol were added to HUVECs for 24 hours or 48 hours and analysed for Erk1/2, p38, Akt phosphorylation and VEGFR2 expression by Western blotting. CTL-control. (**B**) a synergistic effect of kaempferol with VEGF inhibitor PTK787 or FGF inhibitor SU5402 was observed. Compounds were added at 6 hpf and pictured at 52 hpf, anterior is to left and dorsal is to top. Affected ratio is showed in the lower right corner. Full-length blots are presented in Supplementary Fig. S2.

In Fig. 6A, we noted that VEGFR2 was indeed expressed at a lower level when HUVECs were treated with 80 μM kaempferol for 24 hours or 48 hours. ELISA showed that p-Akt level was down-regulated at 20 μM kaempferol treatment in OVCAR-3 cells^23^. Western blot further confirmed that increasing kaempferol concentration reduced p-Akt level without changes in total Akt level. Meanwhile, after treatment by kaempferol at 40 or 80 μM, p-p38 level decreased significantly without affecting the total p38 level (Fig. 6A). Huot *et al.* found that p38 MAPK appeared to mediate migration by enhancing actin polymerization^27^. The reduction of p-p38 MAPK level perhaps explains why kaempferol inhibited VEGF-induced HUVECs migration. Interestingly, p-Erk level, downstream of VEGF and FGF, did not show any observable changes (Fig. 6A).

In zebrafish, PTK787 (a VEGFR2 inhibitor) and SU5402 (an FGFR antagonist) could inhibit the growth of ISVs at 0.63 μM and 1.25 μM, respectively (Fig. 6B). When embryos were co-treated with kaempferol at 10 μM and PTK787 at 0.16 μM or SU5402 at 0.16 μM, ISVs were completely suppressed, same as kaempferol did at 40 μM. However, 10 μM kaempferol, 0.16 μM PTK787 or SU5402 alone treated embryos had no inhibitory effect on ISVs. Overall, this finding suggests that kaempferol inhibits angiogenesis through both VEGF and FGF pathways.

## Discussion

In this study, we performed a pilot screen of 504 fractions on a transgenic zebrafish model to test the utility of the TCM library. One active angiogenesis inhibitor, kaempferol, was identified from fine fractions of *D. versipellis*, but not from crude extracts, showing that our strategy of screening at the fine isolated fraction level indeed increases the success rate of identifying bioactive compounds from TCM.

*D. versipellis* has been used as an herbal medicine for many years. Modern phytochemical studies on *D. versipellis* show that a series of lignans, especially aryl-tetralin lignan podophyllotoxin, are the representative substances^28^. Podophyllotoxin (Compound **13**, Fig. 3), a potent inhibitor of tubulin polymerization, is the dominant component of *D. versipellis*^29^. Its derivative etoposide phosphate (brand names: Eposin, Etopophos, Vepesid, VP-16) is an anticancer drug used in chemotherapy. When podophyllotoxin was tested on the zebrafish model, it was lethal at 10 μM and impaired trunk development at 4 μM, resulting in a small residual trunk on top of the yolk with few blood vessels similar to DYVE (Supplementary Fig. S1, Fig. 2C,D). Podophyllotoxin is rich in *D. versipellis* and very potent, which likely obscured the activity of kaempferol. This explains why the G1 and G2 fractions of *D. versipellis* predominantly show robust toxicity but not antiangiogenic activity. DYVE-D3 is a fine fraction from G3 that exhibits favourable anti-angiogenesis effect. We further obtained five subfractions A~E from DYVE-D3, in which only fraction DYVE–D3B was shown to contain one bioactive compound, kaempferol. This illustrates that kaempferol is the major bio-active compound.

In recent years, flavonoids were found to have many biological activities including antioxidative, anti-inflammatory, gastroprotective, cardioprotective and anticancer effects^30^. Kaempferol is a well-studied natural flavonoid present in many vegetables and fruits, and has been reported to be an antioxidant and antitumor supplement^23^. VEGF and FGF are strong angiogenic factors, which exert their effects via specific binding to cell surface-expressed receptors equipped with tyrosine kinase activity^31^. Luo *et al.* found kaempferol inhibits angiogenesis and VEGF expression in human ovarian cancer cells through both HIF-dependent (Akt/HIF) and HIF-independent (ESRRA) pathways^23^. The decrease of activated Akt most likely promoted apoptosis through Bcl-2 family and caspase-9^32^. They also observed that kaempferol inhibits angiogenesis through an Erk-NFκB-cMyc-p21-VEGF pathway^33^. However, we found p-Erk level did not change significantly in kaempferol treated HUVECs. Recent study demonstrated that VEGFR2 expression is FGF-dependent via activation of Erk1/2 in mice^34^. In our study, we demonstrated that VEGFR2 in HUVECs treated with kaempferol was significantly decreased. In addition, when embryos were co-treated with sub-optimal concentrations of kaempferol and a FGFR inhibitor, the growth of ISVs was abrogated. Our studies, therefore, suggest that kaempferol likely inhibits angiogenesis through regulating both VEGF and FGF pathways, in which direct reduction of VEGF receptor may play a notable role.

## Methods

### Ethics statement

The methods used were carried out in accordance with the approved guidelines. All experimental protocols were approved by Peking University. All animal experiments were approved by the Animal Ethics Committee of Peking University and performed in accordance with the guidelines of Animal Care and Use of Peking University.

### Plant material

The rhizoma of *D. versipellis* was purchased from Guangzhou Qingping Medical Material Market, Guangzhou, China and identified by Professor Danyan Zhang (Guangzhou University of Chinese Medicine) according to the Jiangxi province local standard of Chinese herbal medicine. A voucher specimen (DYVE-2008-GZ-QP) was deposited in the Institute of Traditional Chinese Medicine & Natural Products, Jinan University, Guangzhou, China. Other Chinese medicinal materials were collected in Hubei or Sichuan, China, which were identified by Professor Yahua Zhan, Hubei College of Traditional Chinese medicine or by Professor Hao Zhang, Sichuan University. The specimens are deposited at the Institute of Traditional Chinese Medicine and Natural Products, Jinan University, Guangzhou, China.

### Preparation of fine isolated fractions of *D. versipellis*, compound isolation, and structure identification

Please refer to the Supplementary Extended Experimental Procedures.

### Zebrafish stocks and screening

The transgenic *Tg(kdrl:GRCFP)*^*zn1*^ zebrafish strain was used in this study. Embryos were raised under standard condition and staged according to description by Kimmel *et al.*^35^ Live embryos were placed into 96-well plates, 6 embryos per well, with 200 μL fresh fish water containing 1× Antibiotic–Antimycotic Solution (Mediatech). Fine isolated fraction was added to the embryos at different concentrations according to Supplementary Table S1 at the shield stage of development. Embryos were allowed to grow in the solution up to 3 dpf.

### Cell culture

Kaempferol was 97% purity, as determined by high–performance liquid chromatography. A series of 50 mM stock solution of kaempferol, SU5402 (SML0443, Sigma-Aldrich) and PTK787 (S1101, Selleck) were prepared in DMSO, stored at −20 °C, and then diluted as needed concentrations in cell culture medium. Primary HUVECs (Sciencell, Carlsbad) were cultured in ECM (endothelial cell medium) with 5% (v/v) FBS, 1% (v/v) ECGS, and 1% penicillin–streptomycin (w/v) (Sciencell) at 37 °C under humidified atmosphere of 5% CO_2_. HEK-293 cells were cultured in DMEM (Dulbecco’s Modified Eagle Medium) with 10% (v/v) FBS. The 0.25% (w/v) trypsin with 1 mM EDTA was purchased from Invitrogen. VEGF165 was obtained from GIBCO. Matrigel was purchased from BD Biosciences.

### Cell viability by MTT assay

HUVECs/HEK-293 cells were trypsinized and seeded at 5 × 10^3^ cells/well in 96–well plates. After 24 hours of incubation, HUVECs/HEK-293 cells were treated with different concentrations of kaempferol for 24 hours. Cell proliferation was assessed by MTT.

### Migration assay

HUVECs were allowed to grow into full confluence in 24-well plates, and incubated with complete medium at 37 °C and 5% CO_2_. The HUVECs were then scraped away horizontally in each well using a P100 pipette tip and washed with PBS. ECM supplemented with 0.5% (v/v) FBS was added into well with or without 40 ng/mL VEGF and different concentrations of kaempferol. Three randomly selected views along the scraped line were photographed on each well. After 9–10 hours of incubation, another set of images were taken by the same method. Three independent experiments were performed.

### Tube formation assay

Matrigel (growth factor reduced) was thawed at 4 °C overnight, and each well of a prechilled 96-well plate was coated with 50 μL 1:1 Matrigel, followed by incubation at 37 °C for 40 minutes. HUVECs (15,000 cells) were added in 0.1 mL ECM (supplemented with 0.5% (v/v) FBS and 40 ng/mL VEGF) with various concentrations of kaempferol. After 4 to 6 hours of incubation at 37 °C, 5% CO_2_, tube-like structures were defined as endothelial cord formations that were connected at both ends. The tube-like structures were examined under an inverted microscope.

### Western blots

HUVECs treated with different concentrations of kaempferol were prepared by adding NP-40 lysis buffer with protease and phosphorylase inhibitor (05892791001, 04906845001, Roche). The supernatant was incubated at 95 °C for 20 minutes and was loaded into a 4% stacking, 10% resolving bis-acrylamide gels. Proteins were transferred to polyvinylidene fluoride paper and incubated overnight in primary antibodies: anti-phospho-Akt (#4060, CST), anti-phospho-p44/42 MAPK (#4370, CST), anti-phospho-p38 MAPK (#4511, CST), anti-VEGFR2 (#2479, CST), anti-Akt (#4685, CST), anti-p44/42 MAPK (#4695, CST), anti-p38 MAPK (#8690, CST) and anti-GAPDH (#5174, CST) at a recommended dilution. Primary antibodies were detected using 1:3000 dilution of secondary antibody, Anti-rabbit IgG (#7074, CST), then followed by ECL reagent. Film was scanning and edited by Adobo photoshop CS5.

### Immunostaining

After treated by kaempferol for 48 hours, HUVECs were fixed in 4% PFA for 20 minutes in room temperature. Cell proliferation immunofluorescence was detected by anti-phospho-Histone H3 (#9701, CST) and Alexa Fluor® 488 Donkey anti-Rabbit IgG (A-21206, Life Technologies). Apoptosis in HUVECs was examined following instructions of *In Situ* Cell Death Detection Kit (12156792910, Roche). For DAPI staining, 10000 × stocking buffer was added in PBS and cells were incubated in the dark for 10 minutes after TUNEL assay or phospho-Histone H3 staining.

### Statistical analysis

Experiments were independently repeated at least three times. Then Mean and SEM were calculated. *P* values were calculated using a two-sided un-paired Stutent’s *t*-test.

### Light microscopy

Images were taken with either an Axiovert 200M microscope, or an AxioImager A1 microscope with an AxioCam digital camera (Zeiss), and edited with Photoshop 7.0 (Adobe systems).

## Additional Information

**How to cite this article**: Liang, F. *et al.* Kaempferol Identified by Zebrafish Assay and Fine Fractionations Strategy from *Dysosma versipellis* Inhibits Angiogenesis through VEGF and FGF Pathways. *Sci. Rep.* **5**, 14468; doi: 10.1038/srep14468 (2015).

## Supplementary Material

Supplementary Information
