# Supplementary material for: Kaempferol Identified by Zebrafish Assay and Fine Fractionations Strategy from Dysosma versipellis Inhibits Angiogenesis through VEGF and FGF Pathways
Source: Sci Rep. 2015 Oct 8;5:14468. doi: 10.1038/srep14468 (PMC4597183; doi:10.1038/srep14468)
Supplement: Supplementary Information [file srep14468-s1.doc]

**Kaempferol Identified by Zebrafish Assay and Fine Fractionations Strategy from *Dysosma versipellis* Inhibits Angiogenesis through VEGF and FGF Pathways**

Fang Liang^1,*^, Shengchang Xin^1^,^*^, Hao Gao^2^, Shaodan Chen^2^, Nan Wang^1^, Wei Qin^1^, Hanbing Zhong^3^, Shuo Lin^1,4^, Xinsheng Yao^2^, and Song Li^1^

**From the ^1^**Key Laboratory of Chemical Genomics, School of Chemical Biology and Biotechnology, Peking University Shenzhen Graduate School, Shenzhen, Guangdong, 518055, China. ^2^Institute of Traditional Chinese Medicine & Natural Products, College of Pharmacy, Jinan University, Guangzhou, Guangdong, 510632, China. ^3^Department of Biology, South University of Science and Technology of China, Shenzhen, 518055, China and ^4^Department of Molecular, Cell, and Developmental Biology, University of California, Los Angeles, Los Angeles, CA 90095, USA.

**To whom correspondence should be addressed:** Song Li, Key Laboratory of Chemical Genomics, School of Chemical Biology and Biotechnology, Peking University Shenzhen Graduate School, Shenzhen, Guangdong, 518055 Email: lisong@pkusz.edu.cn; Hao Gao, Institute of Traditional Chinese Medicine & Natural Products, College of Pharmacy, Jinan University, Guangzhou 510632, China, Phone: 86–20–85221559, Fax: 86–20–85221559, Email: tghao@jnu.edu.cn.

^*^ These authors contribute equally.

**SUPPLEMENTARY DATA**

**SUPPLEMENTARY TABLES**

**Table S1. Screening assay of 504 fractions with *Tg(kdrl:GRCFP)^ZN1^* for anti–angiogenesis effects.**

| concentration unit：μg/mL | | | |
| --- | --- | --- | --- |
| TCM plants | ID Number | Concentration | Phenotype |
| *Berberis subacuminata* | BESU | 312 | normal |
|  | BESU-A | 62 | normal |
|  | BESU-A2 | 312 | normal |
|  | BESU-A3 | 312 | normal |
|  | BESU-A4 | 312 | normal |
|  | BESU-A5 | 312 | normal |
|  | BESU-A8 | 12 | dead |
|  | BESU-A6 | 62 | normal |
|  | BESU-A7 | 62 | normal |
|  | BESU-A9 | 12 | normal |
|  | BESU-A10 | 62 | normal |
|  | BESU-A9-1 | 62 | normal |
|  | BESU-A9-2 | 312 | deformity |
|  | BESU-A9-3 | 62 | developmental stalling |
|  | BESU-A10-1 | 312 | normal |
|  | BESU-A10-2 | 62 | normal |
|  | BESU-A10-3 | 312 | deformity |
|  | BESU-B | 312 | normal |
|  | BESU-B1 | 312 | normal |
|  | BESU-B2 | 312 | normal |
|  | BESU-B3 | 312 | deformity |
|  | BESU-C | 312 | normal |
|  | BESU-C1 | 312 | normal |
|  | BESU-C2 | 312 | normal |
|  | BESU-C3 | 312 | ISV development was inhibited |
|  | BESU-C4 | 312 | normal |
|  | BESU-C5 | 312 | normal |
|  | BESU-C6 | 312 | normal |
|  | BESU-C7 | 312 | normal |
|  | BESU-C8 | 312 | normal |
|  | BESU-C9 | 312 | normal |
|  | BESU-C4-1 | 312 | normal |
|  | BESU-C4-2 | 312 | normal |
|  | BESU-C4-3 | 312 | normal |
|  | BESU-C4-4 | 312 | normal |
|  | BESU-C5-1 | 312 | normal |
|  | BESU-C5-2 | 312 | normal |
|  | BESU-C5-3 | 312 | normal |
|  | BESU-C6-1 | 312 | normal |
|  | BESU-C6-2 | 312 | normal |
|  | BESU-C6-3 | 312 | normal |
|  | BESU-C6-4 | 312 | normal |
|  | BESU-C7-1 | 312 | normal |
|  | BESU-C7-2 | 312 | normal |
|  | BESU-C7-3 | 312 | normal |
|  | BESU-C8-1 | 312 | normal |
|  | BESU-C8-2 | 312 | normal |
|  | BESU-C8-2 | 312 | normal |
|  | BESU-C8-4 | 312 | normal |
|  | BESU-D | 312 | normal |
|  | BESU-D1 | 312 | normal |
|  | BESU-D2 | 312 | normal |
|  | BESU-D3 | 312 | normal |
|  | BESU-D4 | 312 | normal |
|  | BESU-D5 | 312 | normal |
|  | BESU-D2-1 | 312 | normal |
|  | BESU-D2-2 | 312 | normal |
|  | BESU-D2-3 | 312 | normal |
|  | BESU-D2-5 | 312 | normal |
|  | BESU-D3-1 | 312 | normal |
|  | BESU-D3-2 | 312 | normal |
|  | BESU-D3-3 | 312 | normal |
| *Dysosma versipellis* | DYVE | 6.25 | the tail did not develop |
|  | DYVE-A | 312 | normal |
|  | DYVE-A1 | 312 | normal |
|  | DYVE-A2 | 1250 | normal |
|  | DYVE-A3 | 1250 | normal |
|  | DYVE-A4 | 78.1 | normal |
|  | DYVE-B | 1250 | normal |
|  | DYVE-B1 | 1250 | developmental stalling |
|  | DYVE-B2 | 312 | normal |
|  | DYVE-B3 | 78.2 | developmental stalling |
|  | DYVE-B4 | 1250 | normal |
|  | DYVE-C | 12.5 | dead |
|  | DYVE-C1 | 1250 | normal |
|  | DYVE-C2 | 312 | normal |
|  | DYVE-C3 | 19.5 | normal |
|  | DYVE-C4 | 19.5 | dead |
|  | DYVE-D | 12.5 | dead |
|  | DYVE-D1 | 12.5 | dead |
|  | DYVE-D2 | 12.5 | dead |
|  | DYVE-D3 | 12.5 | ISV development was inhibited |
|  | DYVE-D4 | 1250 | normal |
|  | DYVE-D5 | 1250 | normal |
|  | DYVE-D6 | 1250 | normal |
|  | DYVE-D7 | 1250 | normal |
| *Gymnadenia conopsea* | GYCO | 312 | normal |
|  | GYCO-A | 62 | normal |
|  | GYCO-A3 | 62 | normal |
|  | GYCO-A4 | 62 | normal |
|  | GYCO-A5 | 312 | normal |
|  | GYCO-A6 | 62 | normal |
|  | GYCO-A7 | 12.4 | normal |
|  | GYCO-A8 | 62 | developmental stalling |
|  | GYCO-A9 | 62 | developmental stalling |
|  | GYCO-A10 | 312 | normal |
|  | GYCO-A10-1 | 312 | normal |
|  | GYCO-A10-2 | 312 | normal |
|  | GYCO-A10-3 | 312 | normal |
|  | GYCO0B | 312 | normal |
|  | GYCO-B1 | 312 | normal |
|  | GYCO-B2 | 312 | normal |
|  | GYCO-B3 | 312 | normal |
|  | GYCO-C | 312 | normal |
|  | GYCO-C1 | 312 | normal |
|  | GYCO-C3 | 312 | normal |
|  | GYCO-C4 | 312 | normal |
|  | GYCO-C5 | 312 | normal |
|  | GYCO-C6 | 312 | normal |
|  | GYCO-C7 | 312 | normal |
|  | GYCO-C7-1 | 312 | normal |
|  | GYCO-C7-2 | 312 | normal |
|  | GYCO-C8 | 312 | normal |
|  | GYCO-C8-1 | 312 | normal |
|  | GYCO-C8-2 | 312 | normal |
|  | GYCO-C9 | 312 | normal |
|  | GYCO-D | 312 | normal |
|  | GYCO-D1 | 312 | normal |
|  | GYCO-D2 | 312 | normal |
|  | GYCO-D2-1 | 312 | normal |
|  | GYCO-D2-2 | 312 | normal |
|  | GYCO-D3 | 312 | normal |
| *Matteuccia orientalis* | MAOR | 62 | normal |
|  | MAOR-A | 62 | deformity |
|  | MAOR-A1 | 312 | normal |
|  | MAOR-A2 | 312 | normal |
|  | MAOR-A3 | 312 | developmental stalling |
|  | MAOR-A4 | 312 | normal |
|  | MAOR-A5 | 62 | normal |
|  | MAOR-A6 | 12.4 | normal |
|  | MAOR-A7 | 62 | normal |
|  | MAOR-A8 | 62 | normal |
|  | MAOR-A9 | 62 | normal |
|  | MAOR-A10 | 312 | normal |
|  | MAOR-A4-1 | 62 | normal |
|  | MAOR-A4-2 | 62 | normal |
|  | MAOR-A4-3 | 312 | normal |
|  | MAOR-A5-1 | 62 | normal |
|  | MAOR-A5-2 | 62 | normal |
|  | MAOR-A5-3 | 62 | normal |
|  | MAOR-A6-1 | 312 | normal |
|  | MAOR-A6-2 | 62 | normal |
|  | MAOR-A6-3 | 62 | normal |
|  | MAOR-A7-1 | 62 | normal |
|  | MAOR-A7-2 | 12.5 | normal |
|  | MAOR-A7-3 | 12.5 | normal |
|  | MAOR-A8-1 | 312 | normal |
|  | MAOR-A8-2 | 62 | normal |
|  | MAOR-A8-3 | 62 | normal |
|  | MAOR-A10-1 | 312 | normal |
|  | MAOR-A10-2 | 62 | developmental stalling |
|  | MAOR-A10-3 | 312 | normal |
|  | MAOR-B | 312 | normal |
|  | MAOR-B1 | 312 | normal |
|  | MAOR-B2 | 62 | normal |
|  | MAOR-B3 | 62 | developmental stalling |
|  | MAOR-B4 | 312 | normal |
|  | MAOR-B5 | 312 | normal |
|  | MAOR-B6 | 312 | normal |
|  | MAOR-B7 | 312 | normal |
|  | MAOR-B8 | 312 | normal |
|  | MAOR-B9 | 312 | normal |
|  | MAOR-B1-1 | 312 | normal |
|  | MAOR-B1-2 | 312 | normal |
|  | MAOR-B1-3 | 312 | normal |
|  | MAOR-B2-1 | 62 | normal |
|  | MAOR-B2-2 | 62 | normal |
|  | MAOR-B2-3 | 12.4 | normal |
|  | MAOR-B3-1 | 312 | normal |
|  | MAOR-B3-2 | 312 | dead |
|  | MAOR-B3-3 | 12.5 | normal |
|  | MAOR-B4-1 | 312 | normal |
|  | MAOR-B4-2 | 312 | normal |
|  | MAOR-B4-3 | 312 | normal |
|  | MAOR-B5-1 | 62 | normal |
|  | MAOR-B5-2 | 312 | normal |
|  | MAOR-B6-1 | 312 | normal |
|  | MAOR-B6-2 | 312 | normal |
|  | MAOR-B7-1 | 312 | normal |
|  | MAOR-B7-2 | 312 | normal |
|  | MAOR-B7-3 | 312 | normal |
|  | MAOR-C | 312 | normal |
|  | MAOR-C1 | 312 | normal |
|  | MAOR-C2 | 62 | normal |
|  | MAOR-C4 | 312 | normal |
|  | MAOR-C5 | 312 | normal |
|  | MAOR-C6 | 312 | normal |
|  | MAOR-C7 | 312 | normal |
|  | MAOR-C8 | 312 | normal |
|  | MAOR-C9 | 312 | normal |
|  | MAOR-C4-1 | 312 | normal |
|  | MAOR-C4-2 | 312 | normal |
|  | MAOR-C4-3 | 312 | deformity |
|  | MAOR-C4-4 | 12.5 | normal |
|  | MAOR-C6-1 | 312 | normal |
|  | MAOR-C6-2 | 312 | normal |
|  | MAOR-C6-3 | 312 | normal |
|  | MAOR-C7-1 | 312 | normal |
|  | MAOR-C7-2 | 312 | normal |
|  | MAOR-C7-3 | 312 | normal |
|  | MAOR-C7-4 | 62 | normal |
|  | MAOR-C7-5 | 312 | normal |
|  | MAOR-D | 312 | normal |
|  | MAOR-D1 | 312 | normal |
|  | MAOR-D2 | 312 | normal |
|  | MAOR-D3 | 312 | normal |
|  | MAOR-D4 | 312 | normal |
|  | MAOR-D2-1 | 312 | normal |
|  | MAOR-D2-2 | 312 | normal |
|  | MAOR-D2-4 | 312 | normal |
| *Osmunda japonica* | OSJA | 312 | normal |
|  | OSJA-A | 312 | normal |
|  | OSJA-A1 | 312 | normal |
|  | OSJA-A2 | 312 | normal |
|  | OSJA-A3 | 312 | normal |
|  | OSJA-A4 | 312 | normal |
|  | OSJA-A5 | 312 | normal |
|  | OSJA-A6 | 312 | normal |
|  | OSJA-A7 | 62 | normal |
|  | OSJA-A8 | 62 | normal |
|  | OSJA-A9 | 312 | normal |
|  | OSJA-A10 | 312 | normal |
|  | OSJA-A2-1 | 312 | normal |
|  | OSJA-A2-2 | 312 | normal |
|  | OSJA-A2-3 | 312 | normal |
|  | OSJA-A9-1 | 156 | thin strip heart |
|  | OSJA-A9-2 | 156 | thin strip heart |
|  | OSJA-A10-1 | 312 | normal |
|  | OSJA-A10-2 | 312 | normal |
|  | OSJA-A10-3 | 312 | normal |
|  | OSJA-B | 312 | normal |
|  | OSJA-B-1 | 312 | normal |
|  | OSJA-B2 | 312 | ISV development was inhibited |
|  | OSJA-B4 | 312 | normal |
|  | OSJA-B5 | 312 | normal |
|  | OSJA-B6 | 312 | normal |
|  | OSJA-B7 | 312 | normal |
|  | OSJA-B8 | 312 | normal |
|  | OSJA-B9 | 312 | normal |
|  | OSJA-C | 312 | normal |
|  | OSJA-C1 | 312 | normal |
|  | OSJA-C2 | 312 | normal |
|  | OSJA-C3 | 312 | normal |
|  | OSJA-C4 | 312 | normal |
|  | OSJA-C5 | 312 | normal |
|  | OSJA-C6 | 312 | normal |
|  | OSJA-C7 | 312 | normal |
|  | OSJA-C8 | 312 | normal |
|  | OSJA-C9 | 312 | normal |
|  | OSJA-C6-1 | 312 | normal |
|  | OSJA-C6-2 | 312 | normal |
|  | OSJA-C6-3 | 312 | normal |
|  | OSJA-C7-1 | 312 | normal |
|  | OSJA-C7-2 | 312 | normal |
|  | OSJA-C8-1 | 312 | normal |
|  | OSJA-C8-2 | 312 | normal |
|  | OSJA-C8-3 | 312 | normal |
|  | OSJA-C8-4 | 312 | normal |
|  | OSJA-C9-1 | 312 | normal |
|  | OSJA-C9-2 | 312 | normal |
|  | OSJA-C9-3 | 312 | normal |
|  | OSJA-C9-4 | 312 | normal |
|  | OSJA-D | 312 | normal |
|  | OSJA-D1 | 312 | normal |
|  | OSJA-D2 | 312 | normal |
|  | OSJA-D3 | 312 | normal |
|  | OSJA-D4 | 312 | normal |
|  | OSJA-D5 | 312 | normal |
|  | OSJA-D2-2 | 312 | normal |
| *Paris chinensis* | PACH | 12.4 | normal |
|  | PACH-B | 12.4 | normal |
|  | PACH-B1 | 312 | normal |
|  | PACH-B2 | 62 | normal |
|  | PACH-B2-1 | 312 | normal |
|  | PACH-B2-2 | 62 | normal |
|  | PACH-B2-3 | 12.4 | normal |
|  | PACH-B3 | 62 | normal |
|  | PACH-B4 | 62 | normal |
|  | PACH-B5 | 12.4 | normal |
|  | PACH-B6 | 12.4 | normal |
|  | PACH-B7 | 12.4 | normal |
|  | PACH-C | 12.4 | normal |
|  | PACH-C1 | 312 | normal |
|  | PACH-C1-5 | 312 | normal |
|  | PACH-C2 | 62 | normal |
|  | PACH-C3 | 312 | normal |
|  | PACH-C4 | 62 | normal |
|  | PACH-C5 | 12.4 | normal |
|  | PACH-C5-1 | 312 | normal |
|  | PACH-C5-2 | 312 | normal |
|  | PACH-C5-3 | 62 | normal |
|  | PACH-C5-4 | 62 | normal |
|  | PACH-C5-5 | 62 | normal |
|  | PACH-C6 | 62 | normal |
|  | PACH-C6-1 | 62 | normal |
|  | PACH-C6-2 | 62 | normal |
|  | PACH-C6-3 | 62 | normal |
|  | PACH-C6-4 | 12.4 | normal |
|  | PACH-C6-5 | 12.4 | normal |
|  | PACH-C7 | 12.4 | normal |
|  | PACH-C7-1 | 62 | normal |
|  | PACH-C7-2 | 62 | normal |
|  | PACH-C7-3 | 62 | normal |
|  | PACH-C7-4 | 12.4 | normal |
|  | PACH-C8 | 12.4 | normal |
|  | PACH-C8-1 | 312 | normal |
|  | PACH-C8-2 | 312 | normal |
|  | PACH-C8-3 | 312 | normal |
|  | PACH-C8-4 | 312 | normal |
|  | PACH-C8-5 | 12.4 | normal |
|  | PACH-C9 | 312 | normal |
|  | PACH-D | 312 | normal |
|  | PACH-D1 | 312 | normal |
|  | PACH-D2 | 312 | normal |
|  | PACH-D2-1 | 312 | normal |
|  | PACH-D2-2 | 312 | normal |
|  | PACH-D2-3 | 62 | normal |
|  | PACH-D3 | 312 | normal |
|  | PACH-D4 | 312 | normal |
| *Panax japonicum* | PAJA | 312 | normal |
|  | PAJA-A | 312 | normal |
|  | PAJA-A2 | 312 | normal |
|  | PAJA-A3 | 312 | normal |
|  | PAJA-A4 | 312 | normal |
|  | PAJA-A5 | 62 | normal |
|  | PAJA-A6 | 62 | normal |
|  | PAJA-A7 | 312 | normal |
|  | PAJA-A8 | 312 | normal |
|  | PAJA-A9 | 312 | normal |
|  | PAJA-A10 | 12.4 | normal |
|  | PAJA-B | 12.4 | normal |
|  | PAJA-B1 | 312 | normal |
|  | PAJA-B2 | 312 | normal |
|  | PAJA-B3 | 312 | normal |
|  | PAJA-B4 | 312 | normal |
|  | PAJA-B5 | 62 | normal |
|  | PAJA-B6 | 2.5 | dead |
|  | PAJA-B7 | 312 | normal |
|  | PAJA-B7-1 | 12.4 | normal |
|  | PAJA-B7-2 | 12.4 | normal |
|  | PAJA-B7-3 | 312 | normal |
|  | PAJA-B8 | 62 | normal |
|  | PAJA-B8-1 | 312 | normal |
|  | PAJA-B8-2 | 312 | normal |
|  | PAJA-B8-3 | 12.4 | normal |
|  | PAJA-B9 | 12.4 | normal |
|  | PAJA-C | 62 | deformity |
|  | PAJA-C1 | 62 | normal |
|  | PAJA-C2 | 312 | normal |
|  | PAJA-C3 | 62 | normal |
|  | PAJA-C4 | 62 | normal |
|  | PAJA-C5 | 62 | normal |
|  | PAJA-C5-1 | 312 | normal |
|  | PAJA-C5-2 | 312 | normal |
|  | PAJA-C5-3 | 312 | normal |
|  | PAJA-C5-4 | 12.4 | normal |
|  | PAJA-C6 | 12.4 | normal |
|  | PAJA-C6-1 | 312 | normal |
|  | PAJA-C6-2 | 312 | normal |
|  | PAJA-C6-3 | 12.4 | normal |
|  | PAJA-C6-4 | 12.4 | normal |
|  | PAJA-C6-5 | 12.4 | normal |
|  | PAJA-C7 | 12.4 | normal |
|  | PAJA-C7-1 | 312 | normal |
|  | PAJA-C7-2 | 312 | normal |
|  | PAJA-C7-3 | 12.4 | normal |
|  | PAJA-C7-4 | 12.4 | normal |
|  | PAJA-C8 | 12.4 | normal |
|  | PAJA-C8-1 | 312 | normal |
|  | PAJA-C8-2 | 312 | normal |
|  | PAJA-C8-3 | 12.4 | normal |
|  | PAJA-C8-4 | 12.4 | normal |
|  | PAJA-C9 | 12.4 | dead |
|  | PAJA-C9-1 | 312 | normal |
|  | PAJA-C9-2 | 12.4 | dead |
|  | PAJA-C9-3 | 62 | normal |
|  | PAJA-C9-4 | 12.4 | thin strip heart |
|  | PAJA-D | 312 | normal |
|  | PAJA-D1 | 312 | normal |
|  | PAJA-D2 | 312 | normal |
|  | PAJA-D2-1 | 312 | normal |
|  | PAJA-D2-2 | 12.4 | normal |
|  | PAJA-D2-3 | 12.4 | normal |
|  | PAJA-D2-5 | 312 | normal |
|  | PAJA-D3 | 12.4 | normal |
|  | PAJA-D3-1 | 312 | normal |
|  | PAJA-D3-2 | 312 | normal |
|  | PAJA-D3-3 | 12.4 | developmental stalling |
|  | PAJA-D3-4 | 12.4 | dead |
|  | PAJA-D4 | 12.4 | normal |
|  | PAJA-D5 | 12.4 | normal |
| *Paeonia veitchii* | PAVE | 312 | normal |
|  | PAVE-A | 312 | normal |
|  | PAVE-A2 | 312 | normal |
|  | PAVE-A2-3 | 312 | normal |
|  | PAVE-A3 | 312 | normal |
|  | PAVE-A4 | 312 | normal |
|  | PAVE-A5 | 62 | normal |
|  | PAVE-A6 | 62 | normal |
|  | PAVE-A7 | 312 | normal |
|  | PAVE-A8 | 312 | normal |
|  | PAVE-A9 | 312 | normal |
|  | PAVE-A10 | 312 | normal |
|  | PAVE-B | 312 | normal |
|  | PAVE-B1 | 62 | normal |
|  | PAVE-B2 | 312 | normal |
|  | PAVE-B3 | 312 | normal |
|  | PAVE-B3-1 | 312 | normal |
|  | PAVE-B3-2 | 312 | normal |
|  | PAVE-B3-3 | 312 | normal |
|  | PAVE-B3-4 | 62 | normal |
|  | PAVE-B4 | 312 | normal |
|  | PAVE-B4-1 | 312 | normal |
|  | PAVE-B4-2 | 62 | normal |
|  | PAVE-B4-3 | 312 | normal |
|  | PAVE-B5 | 312 | normal |
|  | PAVE-B5-2 | 312 | normal |
|  | PAVE-B5-3 | 312 | normal |
|  | PAVE-B6 | 312 | normal |
|  | PAVE-B6-1 | 312 | normal |
|  | PAVE-B6-2 | 312 | normal |
|  | PAVE-B6-3 | 312 | normal |
|  | PAVE-B6-4 | 312 | normal |
|  | PAVE-B7 | 312 | normal |
|  | PAVE-B7-1 | 312 | normal |
|  | PAVE-B7-2 | 62 | normal |
|  | PAVE-B7-3 | 312 | normal |
|  | PAVE-B7-4 | 312 | normal |
|  | PAVE-B8 | 312 | normal |
|  | PAVE-B9 | 312 | normal |
|  | PAVE-C | 312 | normal |
|  | PAVE-C1 | 312 | normal |
|  | PAVE-C2 | 62 | normal |
|  | PAVE-C3 | 312 | normal |
|  | PAVE-C4 | 312 | normal |
|  | PAVE-C4-1 | 312 | normal |
|  | PAVE-C4-2 | 312 | normal |
|  | PAVE-C5 | 312 | normal |
|  | PAVE-C5-1 | 312 | normal |
|  | PAVE-C5-2 | 312 | normal |
|  | PAVE-C5-3 | 312 | normal |
|  | PAVE-C6 | 312 | normal |
|  | PAVE-C6-1 | 312 | normal |
|  | PAVE-C6-2 | 12.4 | normal |
|  | PAVE-C6-3 | 312 | normal |
|  | PAVE-C7 | 312 | normal |
|  | PAVE-C7-1 | 312 | normal |
|  | PAVE-C7-2 | 312 | normal |
|  | PAVE-C7-3 | 312 | normal |
|  | PAVE-C8 | 312 | normal |
|  | PAVE-C8-1 | 312 | normal |
|  | PAVE-C8-2 | 312 | normal |
|  | PAVE-C8-3 | 312 | normal |
|  | PAVE-C9 | 312 | normal |
|  | PAVE-C9-1 | 312 | normal |
|  | PAVE-C9-2 | 312 | normal |
|  | PAVE-C9-3 | 312 | normal |
|  | PAVE-C9-4 | 312 | normal |
| *Vitex trifolia* *var. simplicifolia* | VITR | 312 | normal |
|  | VITR-A | 62 | dead |
|  | VITR-A1 | 312 | normal |
|  | VITR-A2 | 312 | normal |
|  | VITR-A3 | 312 | normal |
|  | VITR-A4 | 312 | normal |
|  | VITR-A5 | 312 | normal |
|  | VITR-A6 | 312 | normal |
|  | VITR-A7 | 62 | dead |
|  | VITR-A8 | 62 | dead |
|  | VITR-A9 | 62 | normal |
|  | VITR-A10 | 62 | normal |
|  | VITR-A6-1 | 312 | deformity |
|  | VITR-A6-2 | 62 | normal |
|  | VITR-A7-1 | 312 | normal |
|  | VITR-A7-2 | 12.4 | normal |
|  | VITR-A8-1 | 12.4 | normal |
|  | VITR-A8-2 | 12.4 | dead |
|  | VITR-A9-1 | 312 | normal |
|  | VITR-A9-2 | 62 | developmental stalling |
|  | VITR-A9-3 | 62 | dead |
|  | VITR-B | 12.4 | normal |
|  | VITR-B1 | 12.4 | normal |
|  | VITR-B2 | 12.4 | normal |
|  | VITR-C | 12.4 | normal |
|  | VITR-C1 | 312 | normal |
|  | VITR-C2 | 12.4 | normal |
|  | VITR-C3 | 312 | normal |
|  | VITR-C4 | 312 | normal |
|  | VITR-C5 | 312 | normal |
|  | VITR-C6 | 312 | normal |
|  | VITR-C7 | 312 | normal |
|  | VITR-C8 | 12.4 | normal |
|  | VITR-C9 | 312 | normal |
|  | VITR-D | 312 | normal |
|  | VITR-D1 | 312 | normal |
|  | VITR-D2 | 312 | normal |
|  | VITR-D3 | 312 | normal |
|  | VITR-D4 | 62 | normal |
|  | VITR-D5 | 12 | dead |
|  | VITR-D2-1 | 312 | normal |
|  | VITR-D2-2 | 312 | normal |
|  | VITR-D2-3 | 62 | normal |
|  | VITR-D3-1 | 312 | normal |
|  | VITR-D3-2 | 312 | normal |
|  | VITR-D3-3 | 312 | normal |

**SUPPLEMENTARY FIGURES AND LEGENDS**

**Figure S1.** **Podophyllotoxin did not inhibit angiogenesis, but severely impaired trunk development**. Upper panel shows the structure of kaempferol and podophyllotoxin. A and C, same embryo treated with 100 μM kaempferol. B and D, same embryo treated with 4 μM podophyllotoxin. A and B, bright field images. C and D, fluorescent images.

**Figure S2. Full-length blots for the data in Figure 6.**

Figure S1


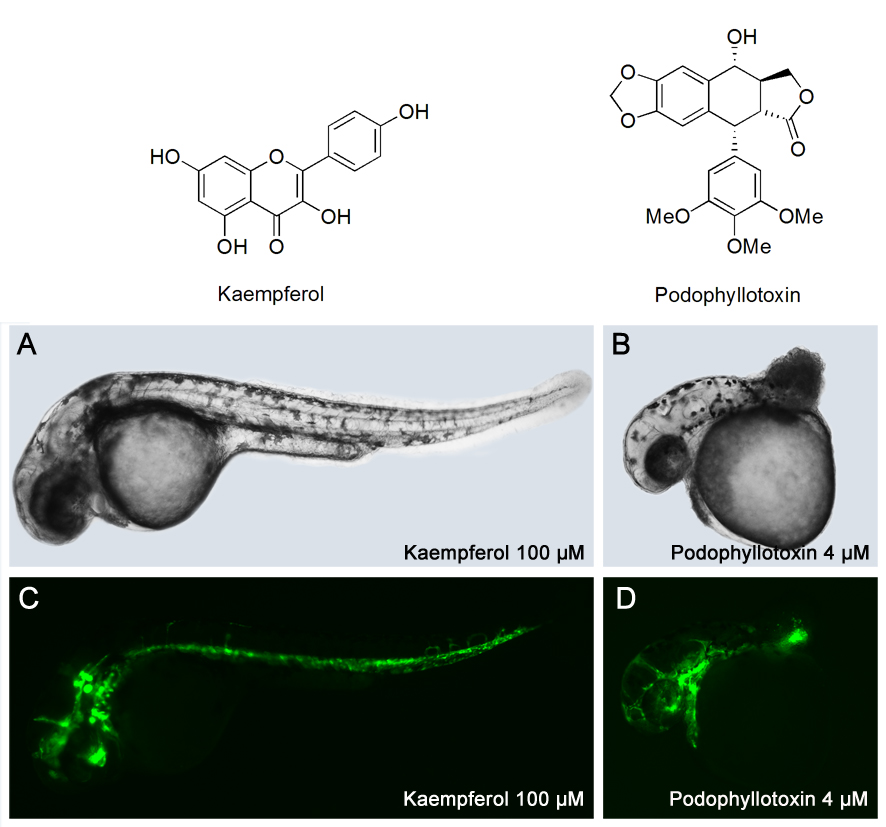


Figure S2


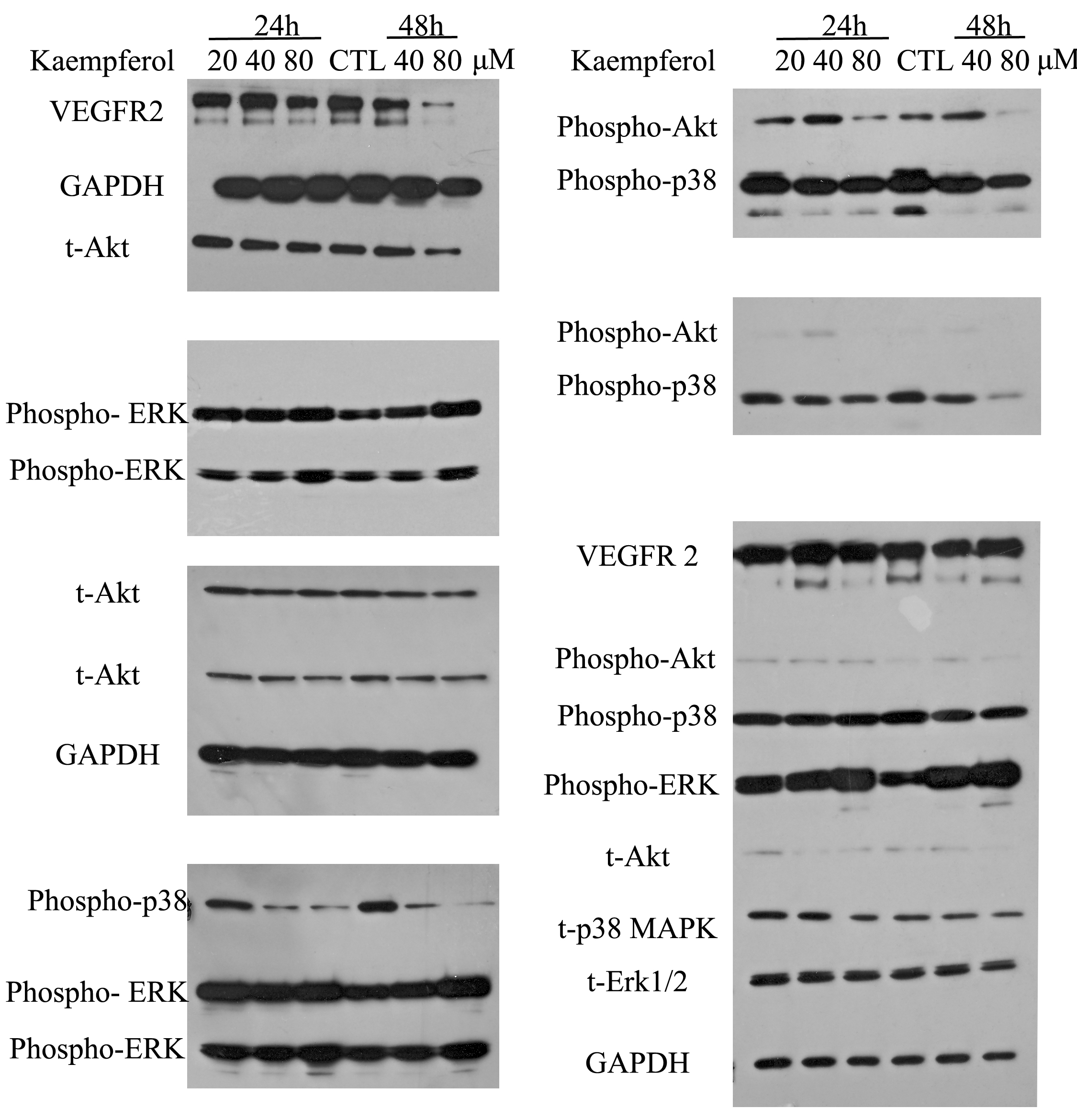


**Extended Experimental Procedure**

**Preparation of fine isolated fractions of the extract of *D. versipellis***

The rattan of *D. versipellis* (0.5 kg) was chopped into small pieces, then extracted with EtOH-H_2_O (60:40, v/v) (1500 mL) by heating the suspension until reflux began (approximately 2 hours). This procedure was repeated two times. After evaporation of the solvent from the combined extract *in vacuo*, the residue DYVE (42.5 g), which was regarded as zero-level fraction, was subjected to column chromatography on macroporous adsorption resin Diaion HP20 (Mitsubishi Chemical, Japan), eluted with a EtOH-H_2_O gradient. The step gradient sequence was 0:100, 30:70, 50:50, and 95:5 (v/v), respectively, with three column volumes (3 × 1000 mL) for each solvent step applied, and the total volume of eluent from each step was collected as one fraction. Then four first-level fractions were yielded: DYVE-A (17.5 g) from the step of 0:100, DYVE-B (5.0 g) from the step of 30:70, DYVE-C (6.25 g) from the step of 50:50, and DYVE-D (5.25 g) from the step of 95:5. For the zero-level fraction and first-level fractions, a portion of 100 mg was conserved for various bioscreenings from each fraction before the subsequent isolation process.

DYVE-A was subjected to medium pressure column chromatography (EYELA, Japan) on ODS (YMC, Japan) and eluted with a MeOH-H_2_O gradient. The step gradient sequence was 0:100, 10:90, 30:70, and 100:0 (v/v), respectively, with three column volumes (3 × 150 mL) for each solvent step applied, and the total volume of eluent from each step was collected as one fraction. Then four second-level fractions were yielded: DYVE-A1 (14.8 g) from the step of 0:100, DYVE-A2 (486 mg) from the step of 10:90, DYVE-A3 (756 mg) from the step of 30:70, and DYVE-A4 (238 mg) from the step of 100:0.

DYVE-B was subjected to medium pressure column chromatography (EYELA, Japan) on ODS (YMC, Japan), eluted with a MeOH-H_2_O gradient. The step gradient sequence was 10:90, 30:70, 50:50, and 100:0 (v/v), respectively, with three column volumes (3 × 150 mL) for each solvent step applied, and the total volume of eluent from each step was collected as one fraction. Then four second-level fractions were yielded: DYVE-B1 (1.46 g) from the step of 10:90, DYVE-B2 (810 mg) from the step of 30:70, DYVE-B3 (1.87 g) from the step of 50:50, and DYVE-B4 (413 mg) from the step of 100:0.

DYVE-C was subjected to medium pressure column chromatography (EYELA, Japan) on ODS (YMC, Japan), eluted with a MeOH-H_2_O gradient. The step gradient sequence was 30:70, 50:50, 70:30, and 100:0 (v/v), respectively, with three column volumes (3 × 150 mL) for each solvent step applied, and the total volume of eluent from each step was collected as one fraction. Then four second-level fractions were yielded: DYVE-C1 (196 mg) from the step of 30:70, DYVE-C2 (680 mg) from the step of 50:50, DYVE-C3 (3.74 g) from the step of 70:30, and DYVE-C4 (1.22 g) from the step of 100:0.

DYVE-D was subjected to column chromatography on silica gel (Qingdao Haiyang Chemical Group Corporation, China), eluted with a CHCl_3_-MeOH gradient. The step gradient sequence was 100:0, 95:5, 9:1, 8:2, 7:3, 1:1, and 0:100 (v/v), respectively, with five column volumes (5 × 150 mL) for each solvent step applied, and the total volume of eluent from each step was collected as one fraction. Then seven second-level fractions were yielded: DYVE-D1 (83 mg) from the step of 100:0, DYVE-D2 (3.86 g) from the step of 95:5, DYVE-D3 (472 mg) from the step of 9:1, DYVE-D4 (323 mg) from the step of 8:2, DYVE-D5 (130 mg) from the step of 7:3, DYVE-D6 (122 mg) from the step of 1:1, and DYVE-D7 (81 mg) from the step of 0:100.

**Compound isolation and structure identification**

NMR spectra were acquired using a Bruker AVANCE 400 NMR spectrometer (400 MHz for ^1^H, 100 MHz for ^13^C). ESI-IT-MS spectra were performed on a Thermo-Finnigan LCQ Advantage MAX mass spectrometer. Analytical HPLC was performed on a Waters HPLC system equipped with 1525 binary HPLC pump, 717 plus autosampler, and 2487 dual λ absorbance detector using an XB-C18 column (4.6 × 250 mm, 5 μm) (Welch Materials, Inc., USA); whereas preparative HPLC was carried out on a Varian instrument (ProStar 210 pump and 325 detector) using an XB-C18 column (21.2 × 250 mm, 5 μm) (Welch Materials, Inc., USA).

Bioguided isolation of the active fraction DYVE-D3 yielded compound **0**. A portion (150 mg) of the active fraction DYVE-D3 was subjected to medium pressure column chromatography (EYELA, Japan) on ODS (YMC, Japan), eluted with a MeOH-H_2_O gradient. The step gradient sequence was 30:70, 50:50, 70:30, 90:10, and 100:0 (v/v), respectively, with four column volumes (4 × 20 mL) for each solvent step applied, and the total volume of eluent from each step was collected as one subfraction. Then five subfractions were yielded: DYVE-D3A (3.6 mg) from the step of 30:70, DYVE-D3B (18.9 mg) from the step of 50:50, DYVE-D3C (78.8 mg) from the step of 70:30, DYVE-D3D (26.7 mg) from the step of 90:10, and DYVE-D3E (9.6 mg) from the step of 100:0. The active component (compound **0**) was obtained from the active subfractions DYVE-D3B by preparative HPLC with MeOH-H_2_O (55:45, v/v) as eluent at 208 nm.

Compounds **1**-**13** were taken from the natural products library constructed by us and their structures were confirmed by NMR and MS data.

Compound **0** (kaempferol): yellow powder; ^1^H NMR (DMSO-*d_6_*, 400 MHz) *δ*: 12.47 (1H, *s*, 5-OH), 10.75 (1H, br. *s*, 7-OH), 10.09 (1H, br. *s*, 4'-OH), 9.35 (1H, br. *s*, 3-OH), 8.04 (2H, *m*, 2', 6'-H), 6.92 (2H, *m*, 3', 5'-H), 6.44 (1H, *d*, *J* = 2.1 Hz, 8-H), 6.19 (1H, *d*, *J* = 2.1 Hz, 6-H); ^13^C NMR & DEPT135 (DMSO-*d_6_*, 100 MHz) *δ*: 175.9 (*s*), 163.9 (*s*), 160.7 (*s*), 159.2 (*s*), 156.2 (*s*), 146.8 (*s*), 135.6 (*s*), 129.5 (*d*), 121.6 (*s*), 115.4 (*d*), 103.0 (*s*), 98.2 (*d*), 93.4 (*d*); +ESI-IT-MS *m/z*: 309 [M + Na]^+^; -ESI-IT-MS *m/z*: 285 [M - H]^-^.

Compound **1** (kaempferol-7-*O*-α-L-rhamnoside): yellow powder; ^1^H NMR (CD_3_OD, 400 MHz) *δ*: 8.10 (2H, *m*, 2', 6'-H), 6.90 (2H, *m*, 3', 5'-H), 6.74 (1H, br. *s*, 8-H), 6.42 (1H, *d*, *J* = 1.8 Hz, 6-H), 5.55 (1H, br. *s*, 1''-H), 4.01 (1H, br. *d*, *J* = 3.3 Hz, 2''-H), 3.83 (1H, *dd*, *J* = 9.4, 3.4 Hz, 3''-H), 3.60 (1H, *m*, 5''-H), 3.47 (1H, *t*, *J* = 9.5 Hz, 4''-H), 1.25 (3H, *d*, *J* = 6.1 Hz, 6''-H); +ESI-IT-MS *m/z*: 455 [M + Na]^+^; -ESI-IT-MS *m/z*: 431 [M - H]^-^.

Compound **2** (kaempferide): yellow powder; ^1^H NMR (DMSO-*d_6_*, 400 MHz) *δ*: 12.44 (1H, *s*, 5-OH), 10.77 (1H, br. *s*, 7-OH), 9.46 (1H, br. *s*, 3-OH), 8.13 (2H, *m*, 2', 6'-H), 7.09 (2H, *m*, 3', 5'-H), 6.45 (1H, *d*, *J* = 2.0 Hz, 8-H), 6.20 (1H, *d*, *J* = 2.0 Hz, 6-H), 3.83 (3H, *s*, 4'-OCH_3_); ^13^C NMR & DEPT135 (DMSO-*d_6_*, 100 MHz) *δ*: 176.0 (*s*), 164.0 (*s*), 160.7 (*s*), 160.4 (*s*), 156.2 (*s*), 146.2 (*s*), 136.0 (*s*), 129.3 (*d*), 123.2 (*s*), 114.0 (*d*), 103.1 (*s*), 98.2 (*d*), 93.5 (*d*), 55.3 (*q*); +ESI-IT-MS *m/z*: 323 [M + Na]^+^; -ESI-IT-MS *m/z*: 299 [M - H]^-^.

Compound **3** (quercetin): yellow powder; ^1^H NMR (DMSO-*d_6_*, 400 MHz) *δ*: 12.46 (1H, *s*, 5-OH), 10.75 (1H, br. *s*, 7-OH), 9.57 (1H, br. *s*, 4'-OH), 9.32 (2H, br. *s*, 3, 3'-OH), 7.68 (1H, *d*, *J* = 2.2 Hz, 2'-H), 7.54 (1H, *dd*, *J* = 8.4, 2.2 Hz, 6'-H), 6.88 (1H, *d*, *J* = 8.4 Hz, 5'-H), 6.40 (1H, *d*, *J* = 2.0 Hz, 8-H), 6.19 (1H, *d*, *J* = 2.0 Hz, 6-H); ^13^C NMR & DEPT135 (DMSO-*d_6_*, 100 MHz) *δ*: 175.8 (*s*), 163.8 (*s*), 160.7 (*s*), 156.1 (*s*), 147.7 (*s*), 146.8 (*s*), 145.0 (*s*), 135.7 (*s*), 121.9 (*s*), 119.9 (*d*), 115.6 (*d*), 115.0 (*d*), 103.0 (*s*), 98.1 (*d*), 93.3 (*d*); +ESI-IT-MS *m/z*: 325 [M + Na]^+^; -ESI-IT-MS *m/z*: 301 [M - H]^-^.

Compound **4** (isorhamnetin): yellow powder; ^1^H NMR (DMSO-*d_6_*, 400 MHz) *δ*: 12.46 (1H, *s*, 5-OH), 10.73 (1H, br. *s*, 7-OH), 9.72 (1H, br. *s*, 4'-OH), 9.42 (1H, br. *s*, 3-OH), 7.76 (1H, *d*, *J* = 2.2 Hz, 2'-H), 7.69 (1H, *dd*, *J* = 8.5, 2.1 Hz, 6'-H), 6.94 (1H, *d*, *J* = 8.4 Hz, 5'-H), 6.47 (1H, *d*, *J* = 2.0 Hz, 8-H), 6.19 (1H, *d*, *J* = 2.0 Hz, 6-H), 3.85 (3H, *s*, 3'-OCH_3_); ^13^C NMR & DEPT135 (DMSO-*d_6_*, 100 MHz) *δ*: 175.8 (*s*), 163.9 (*s*), 160.6 (*s*), 156.1 (*s*), 148.8 (*s*), 147.3 (*s*), 146.6 (*s*), 135.8 (*s*), 121.9 (*s*), 121.7 (*d*), 115.5 (*d*), 111.7 (*d*), 103.0 (*s*), 98.2 (*d*), 93.5 (*d*), 55.8 (*q*); +ESI-IT-MS *m/z*: 339 [M + Na]^+^; -ESI-IT-MS *m/z*: 315 [M - H]^-^.

Compound **5** (astragalin): yellow powder; ^1^H NMR (DMSO-*d_6_*, 400 MHz) *δ*:12.61 (1H, *s*, 5-OH), 8.04 (2H, *m*, 2', 6'-H), 6.88 (2H, *m*, 3', 5'-H), 6.43 (1H, *d*, *J* = 2.0 Hz, 8-H), 6.21 (1H, *d*, *J* = 2.0 Hz, 6-H), 5.46 (1H, *d*, *J* = 7.2 Hz, 1''-H), 3.57 (1H, *m*, 6''-Ha), 3.33 (1H, *m*, 6''-Hb), 3.22 (1H, *m*, 3''-H), 3.19 (1H, *m*, 2''-H), 3.09 (2H, *m*, 4'', 5''-H); ^13^C NMR & DEPT135 (DMSO-*d_6_*, 100 MHz) *δ*: 177.5 (*s*), 164.2 (*s*), 161.2 (*s*), 159.9 (*s*), 156.4 (*s*), 156.2 (*s*), 133.2 (*s*), 130.9 (*d*), 120.9 (*s*), 115.1 (*d*), 104.0 (*s*), 100.9 (*d*), 98.7 (*d*), 93.6 (*d*), 77.5 (*d*), 76.4 (*d*), 74.2 (*d*), 69.9 (*d*), 60.8 (*t*); +ESI-IT-MS *m/z*: 471 [M + Na]^+^; -ESI-IT-MS *m/z*: 447 [M - H]^-^.

Compound **6** (aromadendrin): grey powder; ^1^H NMR (DMSO-*d_6_*, 400 MHz) *δ*: 11.90 (1H, *s*, 5-OH), 10.80 (1H, br. *s*, 7-OH), 9.53 (1H, br. *s*, 4'-OH), 7.31 (2H, *m*, 2', 6'-H), 6.79 (2H, *m*, 3', 5'-H), 5.91 (1H, *d*, *J* = 2.0 Hz, 6-H), 5.86 (1H, *d*, *J* = 2.0 Hz, 8-H), 5.73 (1H, *d*, *J* = 6.3 Hz, 3-OH), 5.05 (1H, *d*, *J* = 11.3 Hz, 2-H), 4.58 (1H, *dd*, *J* = 11.3, 6.1 Hz, 3-H); ^13^C NMR & DEPT135 (DMSO-*d_6_*, 100 MHz) *δ*: 197.8 (*s*), 166.8 (*s*), 163.3 (*s*), 162.6 (*s*), 157.7 (*s*), 129.4 (*d*), 127.5 (*s*), 114.9 (*d*), 100.4 (*s*), 96.0 (*d*), 95.0 (*d*), 82.9 (*d*), 71.4 (*d*); +ESI-IT-MS *m/z*: 311 [M + Na]^+^; -ESI-IT-MS *m/z*: 287 [M - H]^-^.

Compound **7** (naringenin): white powder; ^1^H NMR (DMSO-*d_6_*, 400 MHz) *δ*: 12.14 (1H, *s*, 5-OH), 10.74 (1H, br. *s*, 7-OH), 9.58 (1H, br. *s*, 4'-OH), 7.31 (2H, *m*, 2', 6'-H), 6.79 (2H, *m*, 3', 5'-H), 5.88 (2H, *s*, 6, 8-H), 5.43 (1H, *dd*, *J* = 12.7, 2.9 Hz, 2-H), 3.25 (1H, *dd*, *J* = 17.1, 12.8 Hz, 3-Ha), 2.68 (1H, *dd*, *J* = 17.2, 3.1 Hz, 3-Hb); ^13^C NMR & DEPT135 (DMSO-*d_6_*, 100 MHz) *δ*: 196.3 (*s*), 166.6 (*s*), 163.5 (*s*), 162.9 (*s*), 157.7 (*s*), 128.8 (*s*), 128.3 (*d*), 115.1 (*d*), 101.7 (*s*), 95.8 (*d*), 94.9 (*d*), 78.4 (*d*), 42.0 (*t*); +ESI-IT-MS *m/z*: 295 [M + Na]^+^; -ESI-IT-MS *m/z*: 271 [M - H]^-^.

Compound **8** (hesperetin): white powder; ^1^H NMR (DMSO-*d_6_*, 400 MHz) *δ*: 12.12 (1H, *s*, 5-OH), 10.77 (1H, br. *s*, 7-OH), 9.07 (1H, br. *s*, 3'-OH), 6.93 (1H, *d*, *J* = 8.2 Hz, 5'-H), 6.93 (1H, *d*, *J* = 2.0 Hz, 2'-H), 6.87 (1H, *dd*, *J* = 8.2, 2.0 Hz, 6'-H), 5.90 (1H, *d*, *J* = 2.1 Hz, 8-H), 5.89 (1H, *d*, *J* = 2.1 Hz, 6-H), 5.43 (1H, *dd*, *J* = 12.3, 3.1 Hz, 2-H), 3.77 (3H, *s*, 4'-OCH_3_), 3.19 (1H, *dd*, *J* = 17.2, 12.3 Hz, 3-Ha), 2.71 (1H, *dd*, *J* = 17.2, 3.1 Hz, 3-Hb); ^13^C NMR & DEPT135 (DMSO-*d_6_*, 100 MHz) *δ*: 196.1 (*s*), 166.6 (*s*), 163.4 (*s*), 162.8 (*s*), 147.9 (*s*), 146.4 (*s*), 131.1 (*s*), 117.6 (*d*), 114.0 (*d*), 112.0 (*d*), 101.8 (*s*), 95.8 (*d*), 95.0 (*d*), 78.2 (*d*), 55.7 (*q*), 42.0 (*t*); +ESI-IT-MS *m/z*: 325 [M + Na]^+^; -ESI-IT-MS *m/z*: 301 [M - H]^-^.

Compound **9** (apigenin): grey powder; ^1^H NMR (DMSO-*d_6_*, 400 MHz) *δ*: 12.95 (1H, *s*, 5-OH), 10.72 (1H, br. *s*, 7-OH), 10.41 (1H, br. *s*, 4'-OH), 7.91 (2H, *m*, 2', 6'-H), 6.92 (2H, *m*, 3', 5'-H), 6.76 (1H, *s*, 3-H), 6.47 (1H, *d*, *J* = 2.0 Hz, 8-H), 6.19 (1H, *d*, *J* = 2.0 Hz, 6-H); ^13^C NMR & DEPT135 (DMSO-*d_6_*, 100 MHz) *δ*: 181.7 (*s*), 164.1 (*s*), 163.7 (*s*), 161.4 (*s*), 161.1 (*s*), 157.3 (*s*), 128.4 (*d*), 121.2 (*s*), 115.9 (*d*), 103.7 (*s*), 102.8 (*d*), 98.8 (*d*), 93.9 (*d*); +ESI-IT-MS *m/z*: 293 [M + Na]^+^; -ESI-IT-MS *m/z*: 269 [M - H]^-^.

Compound **10** (chrysin): yellow powder; ^1^H NMR (DMSO-*d_6_*, 400 MHz) *δ*: 12.81 (1H, *s*, 5-OH), 10.89 (1H, br. *s*, 7-OH), 8.05 (2H, *m*, 2', 6'-H), 7.58 (3H, *m*, 3', 4', 5'-H), 6.95 (1H, *s*, 3-H), 6.52 (1H, *d*, *J* = 2.0 Hz, 8-H), 6.22 (1H, *d*, *J* = 2.0 Hz, 6-H); ^13^C NMR & DEPT135 (DMSO-*d_6_*, 100 MHz) *δ*: 181.8 (*s*), 164.4 (*s*), 163.1 (*s*), 161.4 (*s*), 157.4 (*s*), 131.9 (*d*), 130.7 (*s*), 129.1 (*d*), 126.3 (*d*), 105.1 (*d*), 103.9 (*s*), 99.0 (*d*), 94.1 (*d*); +ESI-IT-MS *m/z*: 277 [M + Na]^+^; -ESI-IT-MS *m/z*: 253 [M - H]^-^.

Compound **11** (luteolin): yellow powder; ^1^H NMR (DMSO-*d_6_*, 400 MHz) *δ*: 12.98 (1H, br. *s*, 5-OH), 7.37 (1H, *dd*, *J* = 8.4, 2.2 Hz, 6'-H), 7.36 (1H, *d*, *J* = 2.2 Hz, 2'-H), 6.84 (1H, *d*, *J* = 8.4 Hz, 5'-H), 6.58 (1H, *s*, 3-H), 6.37 (1H, *d*, *J* = 1.9 Hz, 8-H), 6.11 (1H, *d*, *J* = 1.9 Hz, 6-H); ^13^C NMR & DEPT135 (DMSO-*d_6_*, 100 MHz) *δ*: 181.3 (*s*), 166.2 (*s*), 163.7 (*s*), 161.4 (*s*), 157.4 (*s*), 150.8 (*s*), 146.1 (*s*), 120.7 (*s*), 118.9 (*d*), 116.0 (*d*), 112.9 (*d*), 102.9 (*s*), 102.3 (*d*), 99.3 (*d*), 94.1 (*d*); +ESI-IT-MS *m/z*: 309 [M + Na]^+^; -ESI-IT-MS *m/z*: 285 [M - H]^-^.

Compound **12** (genistein): white powder; ^1^H NMR (DMSO-*d_6_*, 400 MHz) *δ*: 12.95 (1H, *s*, 5-OH), 10.84 (1H, br. *s*, 7-OH), 9.57 (1H, br. *s*, 4'-OH), 8.31 (1H, *s*, 2-H), 7.37 (2H, *m*, 2', 6'-H), 6.82 (2H, *m*, 3', 5'-H), 6.38 (1H, *d*, *J* = 2.1 Hz, 8-H), 6.22 (1H, *d*, *J* = 2.1 Hz, 6-H); ^13^C NMR & DEPT135 (DMSO-*d_6_*, 100 MHz) *δ*: 180.2 (*s*), 164.2 (*s*), 162.0 (*s*), 157.6 (*s*), 157.4 (*s*), 153.9 (*d*), 130.1 (*d*), 122.3 (*s*), 121.2 (*s*), 115.0 (*d*), 104.4 (*s*), 98.9 (*d*), 93.6 (*d*); +ESI-IT-MS *m/z*: 293 [M + Na]^+^; -ESI-IT-MS *m/z*: 269 [M - H]^-^.

Compound **13** (podophyllotoxin): white powder; ^1^H NMR (DMSO-*d_6_*, 400 MHz) *δ*: 7.11 (1H, *s*, 5-H), 6.47 (1H, *s*, 8-H), 6.34 (2H, *s*, 2', 6'-H), 5.99 (1H, *s*, O-CH_2_-O), 5.97 (1H, *s*, O-CH_2_-O), 5.76 (1H, *d*, *J* = 7.0 Hz, 4-OH), 4.63 (1H, *dd*, *J* = 9.2, 7.4 Hz, 4-H), 4.49 (1H, *m*, 1-H), 4.48 (1H, *m*, 11-Ha), 4.09 (1H, *m*, 11-Hb), 3.65 (6H, *s*, 3', 5'-OCH_3_), 3.63 (3H, *s*, 4'-OCH_3_), 3.14 (1H, *dd*, *J* = 14.5, 5.3 Hz, 2-H), 2.61 (1H, *m*, 3-H); ^13^C NMR & DEPT135 (DMSO-*d_6_*, 100 MHz) *δ*: 174.7 (*s*), 151.9 (*s*), 146.6 (*s*), 146.4 (*s*), 136.5 (*s*), 136.5 (*s*), 135.0 (*s*), 130.7 (*s*), 109.1 (*d*), 108.4 (*d*), 106.3 (*d*), 101.0 (*t*), 71.0 (*t*), 70.6 (*d*), 59.9 (*q*), 55.9 (*q*), 44.1 (*d*), 43.4 (*d*), 40.1 (*d*); +ESI-IT-MS *m/z*: 437 [M + Na]^+^; -ESI-IT-MS *m/z*: 413 [M - H]^-^.
